# Dual electrochemical sensing of spiked virus and SARS-CoV-2 using natural bed-receptor (MV-gal1)

**DOI:** 10.1038/s41598-021-02029-0

**Published:** 2021-11-26

**Authors:** E. Ghazizadeh, Ali Neshastehriz, Ali Dehghani Firoozabadi, Mohammad Kaji Yazdi, Esmail Saievar-Iranizad, Samira Einali

**Affiliations:** 1 Department of Medical Biotechnology, School of Medicine, Mashhad University of Medical Sciences, Mashhad, Iran; 2 Radiation Biology Research Center, Iran University of Medical Sciences (IUMS), Tehran, Iran; 3 Yazd Cardiovascular Research Center, Shahid Sadoughi University of Medical Sciences, Yazd, Iran; 4 Department of Pediatric Hematologist and Oncologist, Bahrami Children Hospital, Tehran University of Medical Sciences, 25529 Tehran, Iran; 5 Department of Physics, Faculty of Science, University of Tarbiat Modarres, Tehran, Iran

**Keywords:** Biological techniques, Materials science

## Abstract

It has been necessary to use methods that can detect the specificity of a virus during virus screening. In this study, we use a dual platform to identify any spiked virus and specific SARS-CoV-2 antigen, sequentially. We introduce a natural bed-receptor surface as Microparticle Vesicle-Galactins1 (MV-gal1) with the ability of glycan binding to screen every spiked virus. MV are the native vesicles which may have the gal-1 receptor. Gal-1 is the one of lectin receptor which can bind to glycan. After dropping the MV-gal1 on the SCPE/GNP, the sensor is turned on due to the increased electrochemical exchange with [Fe(CN)_6_]^−3/−4^ probe. Dropping the viral particles of SARS-CoV-2 cause to turn off the sensor with covering the sugar bond (early screening). Then, with the addition of Au/Antibody-SARS-CoV-2 on the MV-gal1@SARS-CoV-2 Antigen, the sensor is turned on again due to the electrochemical amplifier of AuNP (specific detection).For the first time, our sensor has the capacity of screening of any spike virus, and the specific detection of COVID-19 (LOD: 4.57 × 10^2^ copies/mL) by using the natural bed-receptor and a specific antibody in the point of care test.

## Introduction

SARS-CoV-2 caused the coronavirus epidemic 2019 (COVID-19), which originated in Wuhan, China^1^. The virus has spread epidemically in other countries, killing many people^2,3^. Early and timely diagnosis of this disease is also essential^4^. Rapid antigen tests are the more tests used to identify COVID-19 due to their high speed and low cost. But, the low sensitivity of them is one of the challenges in these methods^5,6^. In general, RT-PCR, LAMP and RPA methods are the routine methods used to identify COVID-19 which they can use as reliable methods in laboratories^7,8^. Although, the time-consuming and requires extensive laboratory equipment are the problems of this method^9^. Biosensor can be useful as the tools with high power and sensitivity and a reasonable cost, with usability in a point-of-care test^10^. Because the spikes of the SARS-CoV-2 have a glycan structure^11^, the basics of our screening are about the identification of glycan. So far, many nanomaterials are used as platforms to detect viral particles^12,13^. On the other hand, many artificial receptors have been used to identify the glycan ligands^14–16^. Galectins are the lectin receptors which can act as a receptor/ligand on the surface of exosomes or microparticle vesicles (MV)^17^. Most studies about SARS-CoV-2 have shown that the spike is connected via the CTD-S1 domain with ACE-2. But, a recent study showed the effect of the NTD domain on the binding with GM1 ganglioside to maintain stability^18,19^. Our results were demonstrated the probable effect of NTD-S1 domain binding with glycan by electrochemical behaviors via introducing the MV-gal1 as a natural receptor-bed with the electrochemical properties, for the first time. Ghazizadeh et al. showed the electrochemical properties of exosomes as a natural bed. They used of the synthetic receptor (p19 protein) to sense of RNA^20^. Here, we use as natural bed-receptor without any synthetic processes. Gal-1 is a natural receptor on the natural bed (MV) which is extracted of marrow-derived mesenchymal stem cells to general screening of every spiked virus. To specific detection of SARS-CoV-2 Antigen, we used as Au@Antibody-SARS-CoV-2 spike to bind the SARS-CoV-2 virus. So, with the addition of MV-gal1 on the SCPE/GNP, the impedance increased (sensor = ON) due to exchange of charge of gal-1 with [Fe(CN)_6_]^−3/−4^. Then, with the addition of the inactive SARS-CoV-2 on MV-gal1/SCPE-GNP, the impedance decreased by covering of the glycosylation bond. Dropping the Au@Anti-SARS-CoV-2 spike caused to turn on the sensor by electrochemical amplifier of AuNP, again. As a result, we reported a natural bed/receptor (MV-gal1) with Au@Anti-SARS-CoV-2 spike to double sensing of SARS-CoV-2 Antigen with high sensitivity in ~ 5 min.

## Materials and methods

### Materials

All of the materials as analytical grade potassium ferrocyanide, potassium ferricyanide, sulfuric acid, hydrogen peroxide, sodium chloride, and potassium chloride were prepared from Novin Tech Company, IRAN. We used as Virus Transport Medium (VTM) from Nedashimi Co, IRAN. This is a liquid media for the transport of specimens to the laboratory for transport of viruses (including COVID19). We also used as SCPE/GNP which is functionalized with gold nanoparticles on the ceramic substrate were purchased from DropSens Inc. Gold Nano Particle-Carbon (GNP-carbon) working electrode; a carbon counter electrode and a silver reference electrode are components of the electrode. Electrochemical impedance spectroscopy (EIS) and Differential pulse voltammetry (DPV), were evaluated by SP-300 Instruments (SP-300) Texas, USA. DPV was done in the presence of 1 mM [Fe(CN)_6_]^−3/−4^ in phosphate buffer saline in the potential window − 0.4 V to + 0.4 V at a scan rate 50 mV s^−1^, and impedance measurement was done between 100 kHz to 1 Hz of [Fe(CN)_6_]^−3/−4^ in phosphate buffer saline pH = 7.4. ZSimpWin 3.22 Software (Princeton Applied Research) was used for measuring the EIS spectra with the help of equivalent circuit using, and the data were presented in Nyquist plots. AFM was done for the analysis of the surface roughness on a Dimension 3000 instrument (Digital Instruments/Aveco Science). TEM images were done by TecnaiG220 instruments from FEI Company, Hillsboro, USA. We determinate the particle sizes and zeta potentials by Horiba nanoparticle size analyzer, Malvern Nano SZ-100 at wavelength 532 nm.

### Experimental autoimmune encephalomyelitis (EAE) induction

We purchased the female C57BL/6 mice at 6–8 weeks old from Pasteur Institute, Iran. Animals were kept under pathogen-free conditions at the animal house of IPIU (Institute Physiology of IRAN University). They were treated according to the National Institute of Health Guide for Care and Use of Laboratory Animals. We have done the EAE induction based on the previous protocol^21^.

### Isolation and characterization of microparticle vesicles (MSC)

Marrow-derived mesenchymal stem cells (MSC) were harvested from the Tibia and Femurs of healthy C57BL/6 mice by flushing method. Cells were taken to plate in T75 flasks using low-glucose Dulbecco’s Modified Eagle’s Medium (LG-DMEM; Invitrogen, Carlsbad, CA), after centrifugation at 1500 rpm for 5 min in Hank’s Balanced Salt Solution buffer (HBSS; Invitrogen, Carlsbad, CA). Then we plated the cells containing 15% fetal bovine serum (FBS; Invitrogen, Carlsbad, CA) and antibiotics. Non-adherent cells were removed after 6 h incubation at 37 °C and humidified 5% CO_2_. When cultured showed > 70% confluence, adherent cells were gathered using 10 min with incubation at 37 °C with 0.05% trypsin (Invitrogen, Carlsbad, CA) solution containing 0.02% ethylene diamine tetraacetic acid (EDTA; Sigma-Aldrich, St. Louis, MO) and washed twice with phosphate buffer saline. Harvested cells were transferred into the T25 flasks for sub-culturing^21^. We used the 3rd passage of MSCs (adherent cells) for flow cytometry analysis. Surface expression of stem cell markers characterized using anti-mouse monoclonal antibodies against CD90-PE, and CD73-FITC (all purchased from eBioscience, San Diego, CA). Flow cytometric analyses were performed using a PAS flow cytometer (Partec GmbH, Germany). Cell Quest software was used for data analysis.

### Isolation and characterization MV

Isolation of MVs were done as previously published protocol^22^. After collecting the supernatant of MSC culture, they were centrifuged at 300×*g* for 10 min, 1000×*g* for 20 min, and 10,000×*g* for 30 min. Then, the final centrifuged supernatant was ultra-centrifuged at 100,000*g* for 2 h in the ultracentrifuge (Beckman coulter optima TMXL-100K ultracentrifuge. The pelleted MV was washed in saline and again centrifuged at 100,000*g* for 2 h. The suspension pellet was quantified by Bradford assay (Sigma-Aldrich, St. Louis, MO).

### Flow cytometry analysis for MV/gal-1

The MVs (40 µg) were incubated with 4 µm diameter aldehyde/sulfate latex beads (Invitrogen, Carlsbad, CA), for 4 h at 37 °C with gentle mixing. We use as 100 mM glycine to fill reactive sites on the beads’ surface to prevent the coupling reaction was stopped. To form pellet MV-coated beads, the mixture was centrifuged at 3000×*g* for 20 min^23^. Then, the suspension of the pellet in phosphate buffer saline was occurred and then washed three times. MV-coated beads were stained using specific antibodies to CD9 FITC, CD63 Biotin followed by streptavidin PE and Anti-GAL1 FITC (MyBiotech Co).

### Virus culture

The infection of corona virus was done in a biosafety level 3 laboratory at Pasteur institute. We use as an African green monkey kidney Vero E6 cells with a clinical isolate of SARS-CoV-2 (https://wwwnc.cdc.gov/travel/notices/covid-4/coronavirus-iran). We collected the culture medium containing mature infectious virus (virus medium), and titration were done by plaque assay. Live virus was inactivated by heating at 100 °C for 15 min and was stored at − 80 °C for further use.

### Clinical sample preparation

The clinical samples used in this were collected who Suspicious patients referred to Emad laboratory were used. They provided written informed consent as registration number: EHW 2020-04-07-507). In addition to this laboratory, ethical committed of Iran medical university confirmed that all experiments were performed in accordance with relevant guidelines and regulations with registration code as: 99-1-6-8-17943) Detailed and clinical information of the participants is given in Table S2, as follows human guidelines. Nasopharyngeal swabs from COVID-19 patients and healthy subjects were stored in VTM (NedaShimi, IRAN). Viral copy number was determined by real-time RT-PCR. Clinical samples were inactivated by heating at 100 °C for 10 min and were stored at − 80 °C for further use.

### Preparation of MV-gal1/SARS-CoV-2 antigen on the SCPE-GNP

Immobilization of MV-gal1 was done by dropping 5.2 µL of MV-gal1 solution in 50 mM phosphate buffered saline (phosphate buffer saline, pH 7.4) onto the SCPE/GNP and incubated overnight at 4 °C. After incubation, excess MV-gal1 was removed by the phosphate buffer saline. Following rinsing, 50 µL of blocking solution (1% BSA in phosphate buffer saline for 1 h) was added onto the electrode surface to prevent the nonspecific binding and incubated at 4 °C. Then we use as SARS-CoV-2 Antigen as SARS-CoV-2 Antigen Protein stock (ProSci Incorporated, Co) which was diluted to 100 fold a 5 µL of this diluted solution was dropped on the MV-gal1/SCPE and incubated overnight at 4 °C. 3% BSA was added to the antibody solutions for blocking and minimize the non-specific absorption (NSA). Then, electrochemical tests were done at every stage.

### Bioconjugation of gold nanoparticle to Anti-SARS-CoV-2 spike

A mixture of 100 µL of Anti-SARS-CoV-2 spike (50 µg/mL in 5 mM KH_2_PO_4_, pH 7.5) (MyBiotech Co) and 700 µL of 0.1% Au nanoparticle solution was prepared a kept for 50 min at room temperature. We add 50 µL of 1% PEG in 5 mM KH_2_PO_4_ solution (pH = 7.5) and 100 µL of 10% BSA in 50 mM KH_2_PO_4_ solution (pH 9.0) to block any uncovered surface on the AuNPs. The AuNP conjugated Anti-Cov-2 (Au/Anti-SARS-CoV-2 spike) was then collected via centrifugation (8000*g* for 15 min at 4 °C). Au/Anti-SARS-CoV-2 spike were suspended in 1 mL of preservation solution (1% BSA, 0.05% PEG 20000, 0.1% NaN_3_ and 150 mM NaCl in 20 mM Tris HCl buffer, (pH = 8.2), and centrifuged again to collect the Au/Anti-SARS-CoV-2 spike. and stored as stock solution.

### Sandwiched Au/Anti-SARS-CoV-2 spike on the MV-gal1/SARS-CoV-2 antigen protein SCPE-GNP

The Au/Anti-SARS-CoV-2 spike stock solution was diluted to tenfold and 6 µL of this diluted solution was dropped onto the MV-gal1/SARS-CoV-2 Antigen Protein. After incubation for 30 min at room temperature, the surface was left for 1 h and washed with blank phosphate buffer saline. So, the electrochemical tests were done, again.

## Results

### MSC and MVs characterization

**Figure 1:**
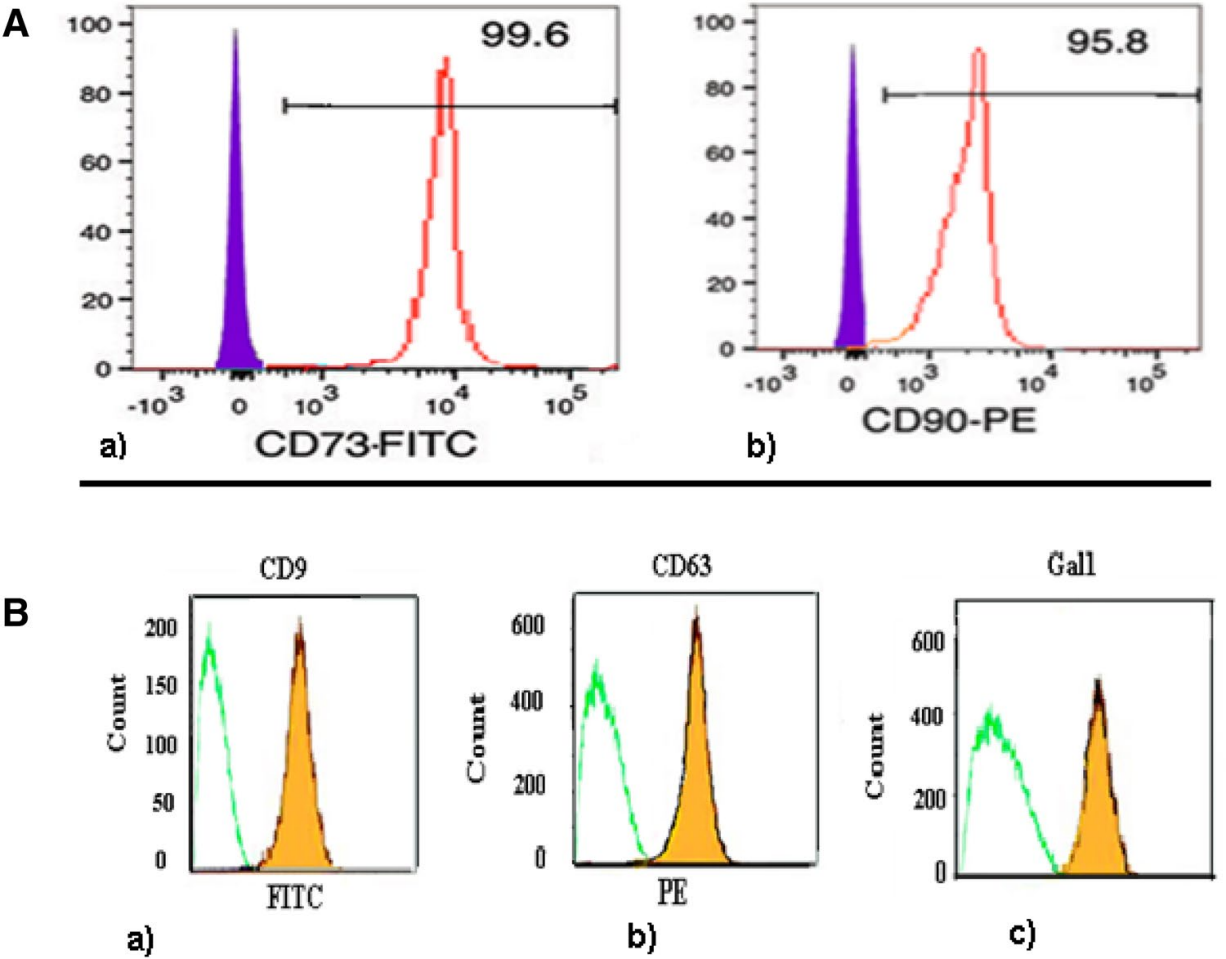
(**A**) Characterization of mouse bone marrow-derived mesenchymal stem cells. Flow cytometry analyses of cell surface markers showed that cultured cells were positive for mesenchymal stem cell markers of CD90 (99.6%) (a), CD73 (95.8%) (b). (**B**) Flow cytometry analyses of MVs surface markers. MVs coated on sulfate/aldehyde latex beads were positive for the surface expression of CD9 (95.6%) (a), CD81 (94.8%) (b), Gal-1 (84.8%) (c).

There was a homogenous population of MSCs which obtained from C57BL/6 mice after 3 passages in vitro. Flow cytometry analyses show the expression of CD90 and CD73 (Fig. 1A). Analyses of MVs by electron microscope showed the presence of nano-sized vesicles which size of them at the range of 50 and 200 nm (Fig. S1). In MVs, flow cytometry analyses were positive for the expression of surface markers of CD9, C63, and Gal-1 (Fig. 1B).

### Fabrication and characterization of bio sensing MV-gal1@SARS-CoV-2 Antigen@Au/Anti-SARS-CoV-2 spike on the SCPE-GNP

**Figure 2:**
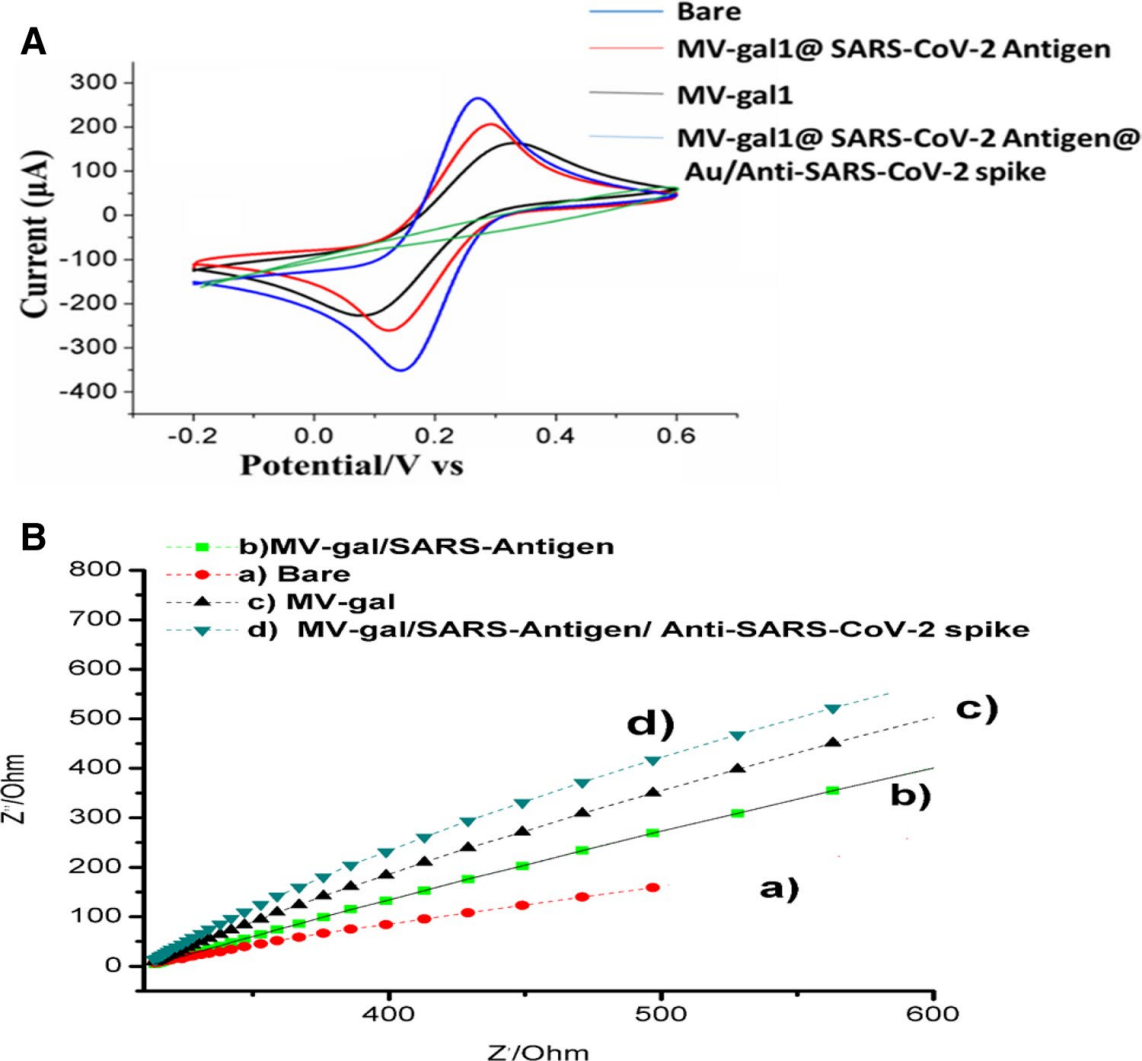
(**A**,**B**) DPV and EIS behaviors about and EIS images about fabrication of MV-gal1@SARS-CoV-2 Antigen@Au/Anti-SARS-CoV-2 spike on the SCPE-GNP. Data recorded at the scan rate 50 mV s^−1^ in phosphate buffer (pH 7.4) containing 1 mM [Fe(CN)_6_]^−3/−4^.

Immobilization of every material on the GNP-SCPE showed the different differential pulse voltammetry in the presence of 1 mM in phosphate buffer saline (Fig. 2A,B). At first, the Bare gold electrode showed the ΔE (Epa − Epc) 75 mV in the [Fe (CN)_6_]^−3/−4^ redox probe. Attachment of the MV-gal1 on the GNP-SCPE was done by direct drop casting^24^ and showed the peak currents decreased from 14.3 to 13.9 µA and the ΔEp increased from 75 to 83 mV, due to the increasing the electrochemical reaction with the receptors on the MV with the [Fe(CN)_6_]^−3/−4^ redox probe. A recent study of Ghazizadeh, showed that exosomes have the electrochemical properties on the SCPE-GNP^20,25^]. Our results about MV are overlap with considering they are larger microparticles with the similar exosomal properties. Adding the SARS-CoV-2 Antigen on the MV-gal1/SCPE-GNP shows that the peak currents increased from 13.9 to 15.4 µA and the ΔEp decreased from 83 to 73 mV, due to covering the glycosylation bond by gal1 and glycan of spike of SARS-CoV-2. A recent study of Kajiyazdi was showed that covering the glycosylation bonds when lectin used as a synthetic receptor to identify the glycan on the tumor cells of AML disease^26,27^. Our results indicate the possible connection of the gal1 with NTD-domain (Glycan domain) of the SARS-CoV-2 Antigen by increasing the current peak, which it causes to the reduction of electrochemical exchanges with the [Fe(CN)_6_]^−3/−4^ redox probe, too. At the end, adding the Au@Anti-SARS-CoV-2 spike on the MV-gal1/SARS-CoV-2 Antigen/SCPE-GNP shows the decreasing in peck current, again (15.4–14.2 µA) and ΔEp increased from 73 to 89 due to amplify reactions of AuNP with [Fe(CN)_6_]^−3/−4^ redox pro.

In fact, we used in the study of direct adsorption of AuNP with Anti-SARS-CoV-2 spike to form the Au@Anti-SARS-CoV-2^28^. In order to ensure the binding of AuNP to the Anti-SARS-CoV-2 spike, we used MV-gal1@SARS-CoV-2 Antigen@Anti-SARS-CoV-2 (Without AuNP as control) in the electrochemical reaction of DPV, which showed a noticeable decrease in MV-gal1@SARS-CoV-2 Antigen@Au/Anti-SARS-CoV-2 relative to MV-gal1@SARS-CoV-2 Antigen@Anti-SARS-CoV-2 (73–65 ΔEp). These results indicate the binding and effect of gold nanoparticles on antibodies and increase in electrochemical exchanges (Fig. S5).

**Figure 3:**
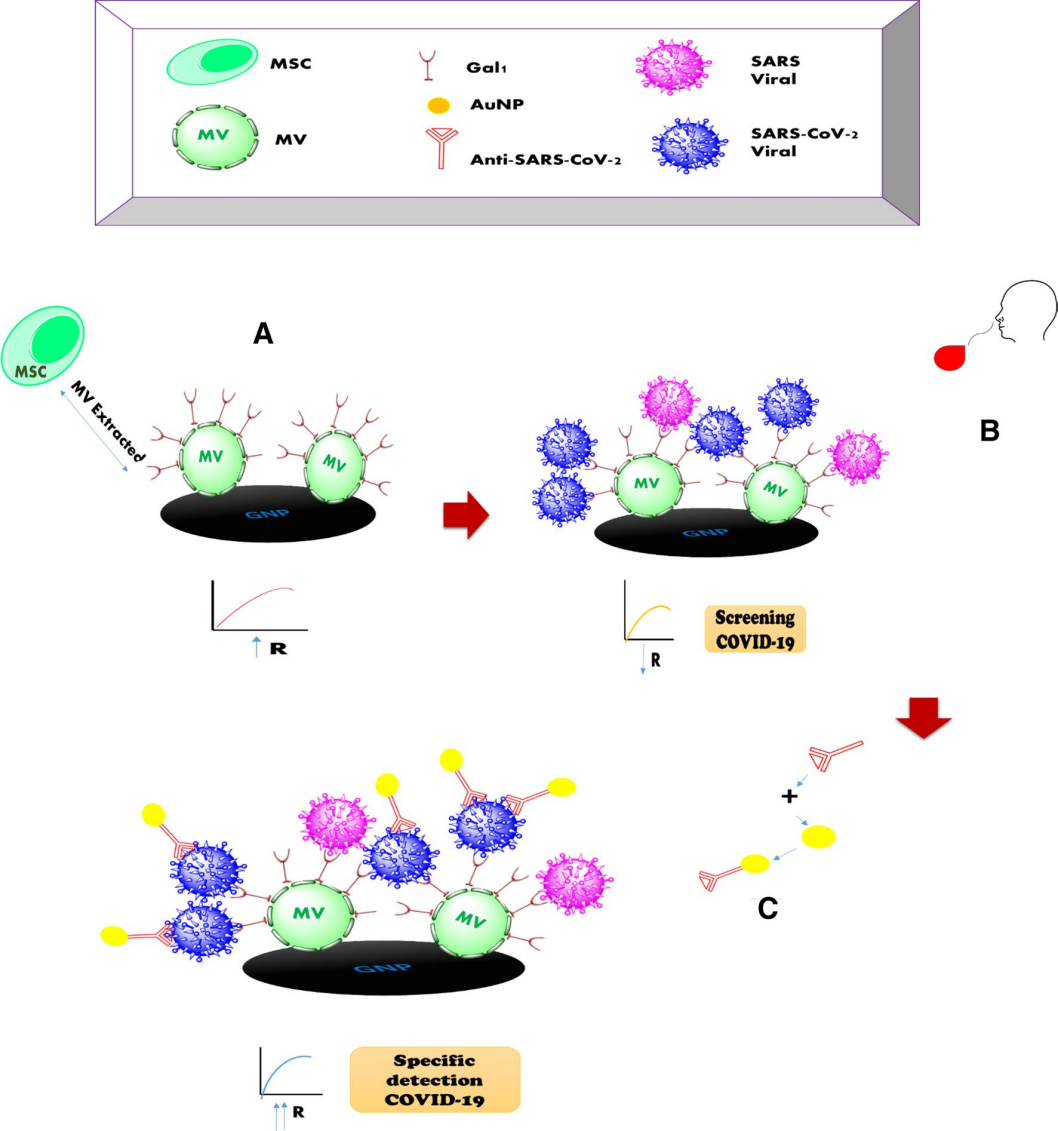
Schematic schema for the stages of screening and specific detection of COVID-19 based on electrochemical biosensor. (**A**) Immobilization of MV-gal1 on the SCPE-GNP. (**B**) Dropping the SARS-CoV-2 on the MV-gal1-SCPE-GNP to screening the spiked COVID-19. (**C**) Dropping the Au/Anti-SARS-CoV-2 spike On the MV-gal1@SARS-CoV-2 Antigen to specific detection of COVID-19.

**Figure 4:**
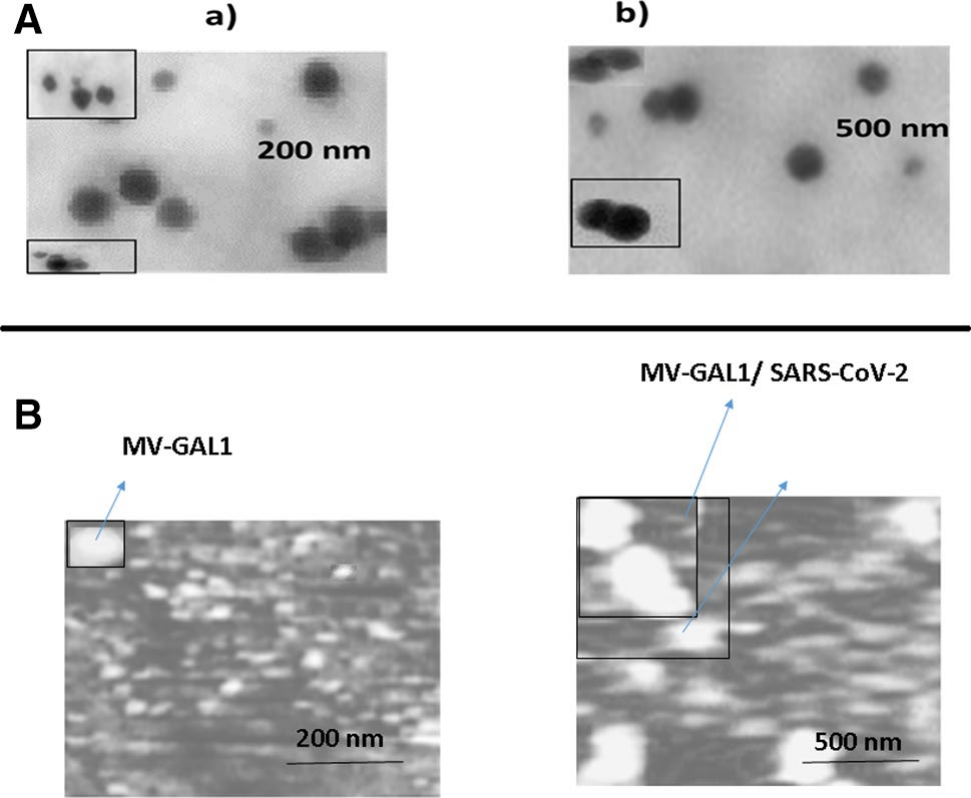
(**A**) TEM images of MV-gal1 (a) and MV-gal1@SARS-CoV-2 virus (b) to show the modified SCPE/GNP. (**B**) AFM images when adding of MV-gal1 (a) and MV-gal1@SARS-CoV-2 virus (b) were occurred on the SCPE/GNP.

In research of Dianyun showed that gold nanoparticle (AuNP) can act as an electrochemical amplifier to detection human chorionic gonadotropin (hCG) which it is used as a label with the second AB^29^. Not only, adding the Au@Anti-SARS-CoV-2 spike on the MV-gal1@SARS-CoV-2 Antigen show the specific SARS-CoV-2 Antigen (specific detection) with the increase in the electrochemical reaction of AuNP with Fe(CN)_6_]^−3/−4^ probe, but only it can prove the connection of MV-gal1 with glycans of SARS-CoV-2 which happened in first step, too. So at this point, it may confirm the connection of gal-1 on the MV with the spike of SARS-CoV-2 based on the NTD-domains. We also verified the effect of bonding of glycans in attachment with receptors based on the electrochemical reactions. We use as the EIS to identify the modified electrode with surface properties. The impedance behaviors of the respective layers are reported in Fig. 2B. Two equivalent circuits viz., Rs(Qdl(RCTW)) and Rs(Qdl(RCTW)(CRL)) were used as model to show the impedance data. Rs(Qdl(RCTW)) will use for further analysis if the Rs(Qdl(RCTW)(CRL)) circuit does not fit well with all surfaces studied. So, Rs shows the solution resistance; Qdl and R_*CT*_ are capacitance (constant phase element) and charge transfer resistance of the gold electrode respectively; RL is the layer resistance and W is Warburg element. In corroboration with the DPV results, the R_*CT*_ value is 5.32 × 10^4^ Ω cm^−2^ for the bare gold electrode. R_*CT*_ value increased to 6.58 × 10^4^ Ω cm^−2^ when adding the MV-gal1 and decreased to 5.83 × 10^4^ Ω cm^−2^ with added SARS-CoV-2 Antigen on the MV-gal1. At the end, R_*CT*_ value increased again to 7.32 × 10^4^ Ω cm^−2^ when the Au@Anti-SARS-CoV-2 spike was dropped on the modified surface. The schematic figure of every steps of modified sensor were described in Fig. 3. The AFM image of the MV-gal1/SCPE-GNP surface becomes smoother following the hybridization by adding SARS-CoV-2 virus compare to rough surface of the MV-gal1 because of higher sizer materials (adding SARS-CoV-2 viral) than MV-gal1 with providing the electrostatic blockade for the linear diffusion of the [Fe(CN)_6_]^−3/−4^ directly to the electrode surface (Fig. 4B(b)). To identify the interaction of each component on the modified SCPE/GNP sensor, TEM images have been used. Figure 4A panel (a) shows the TEM images of the modified MV-gal1 at higher magnification, ~ 300 nm. The interaction of SARS-CoV-2 virus on MV-gal1 has shown with the increased size of the ~ 650 nm. (Fig. 4A(b).

### Dual sensing of SARS-CoV-2 antigen protein

**Figure 5:**
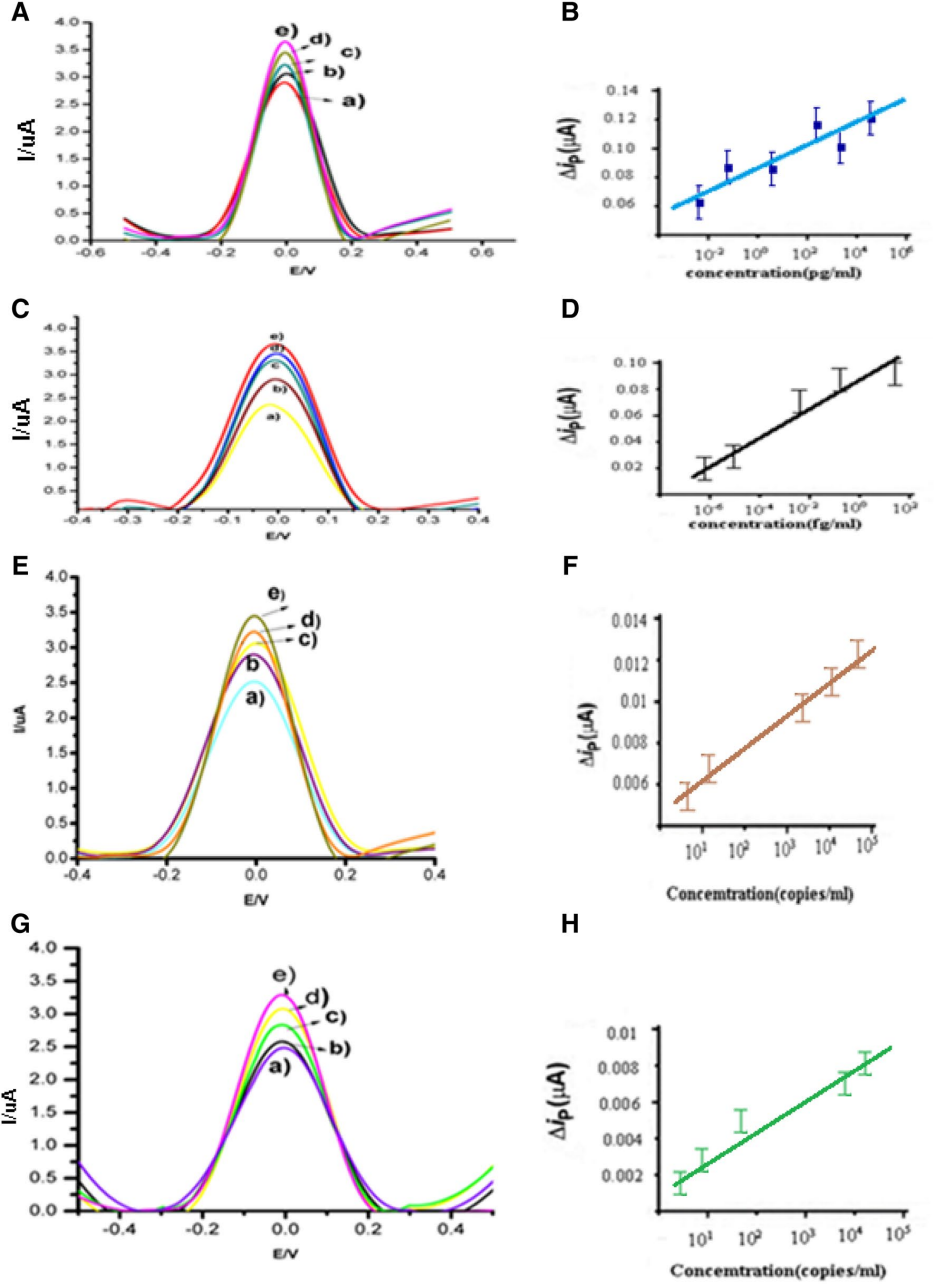
The performance of the sensor for screening and specific detection of SARS-CoV-2 in vitro and clinical samples using DPV measured at a scan rate 50 mV s^−1^ in phosphate buffer (pH 7.4). (**A**) Screening of SARS-CoV-2 Antigen based on DPV images using the 1 µg/mL (e) to 500 fg/mL (a) on the MV-gal1/SCPE-GNP in vitro. (**B**) A calibration plot of the current density vs log concentration of SARS-CoV-2 Antigen. (**C**) Specific detection of SARS-CoV-2 Antigen based on DPV images using the 1 1 fg/mL (e) to 250 pg/mL. (**D**) A calibration plot of the current density vs log concentration of SARS-CoV-2 Antigen, in vitro. (**E**) SARS-CoV-2 Virus screening from clinical samples based on DPV. (**F**) A calibration plot of the current density vs log concentration of SARS-CoV-2 virus (copies/mL). (**G**) Detection of specific SARS-CoV-2 Virus from clinical samples based on DPV. (**H**) A calibration plot of the current density vs log concentration of SARS-CoV-2 virus (copies/mL).

To investigate the performance of the MV-gal1/SCPE-GNP sensor, we evaluated the response of the sensor to SARS-CoV-2 Antigen Protein. First, we showed the LOD of sensor for spike protein when it connected to MV-gal1. Our devise sensed to 500 fg/mL of SARS-CoV-2 spike protein in phosphate buffer saline (Fig. 5A). The j value increases linearly with increasing the concentration of SARS-CoV-2 spike protein ranged from 1 µg/mL to 500 fg/mL. A regression equation of y = 10.973x + 30.456 (R^2^ = 0.976) was obtained, where y is the j value in μA cm^−2^ and x is the logarithmic concentration of SARS-CoV-2 spike protein in µg/mL. The sensor responded the LOD (1 fg/mL) with lower sensing of SARS-CoV-2 spike protein in phosphate buffer saline when the Au@Anti-SARS-CoV-2 spike was added on the SCPE-GNP electrode. A regression equation of y = 12.763x + 30.456 (R^2^ = 0.976) was obtained, where y is the j value in μA cm^−2^ and x is the logarithmic concentration of SARS-CoV-2 spike protein in fg/Ml (Fig. 5C,D). So, it can indicate high sensitivity and specificity for detection of the SARS-CoV-2 spike antigen. Also, it can be proved that the connection of MV-gal1 with glycan of SARS-CoV-2 spike protein which it can cause the connection of Au@Anti-SARS-CoV-2 spike via another specific part of SARS-CoV-2 spike protein. To diagnosis of COVID-19 is performed using nasopharyngeal swabs suspended in transport medium (VTM). So, we used as our sensor to detection of SARS-CoV-2 Antigen protein in Universal Transport Medium (UTM). However, the presence of various reagents such as salts and non-specific factors can affect the performances of the sensor, but our sensor can sense SRS-CoV-2 spike proteins in 0.01 × VTM with starting from a concentration of 1 µg/mL when it attached to the MV-gal1/SCPE-GNP sensor and 500 fg/mL when adding of the Au@Anti-SARS-CoV-2 spike was occurred (Fig. S2A,B). So, our device can screen and specific detection of the COVID-19 samples in two steps without any preparation or preprocessing.

### Sensing of SARS-CoV-2 virus from clinical samples

Our sensor was used to show the functionality of COVID-19 in clinical samples (Fig. 5). So, we collected the nasopharyngeal swab specimens from COVID-19 patients (Emad laboratory) and normal subjects and stored them in VTM (Table S1). We optimize the nasopharyngeal swab samples relate to normal subjects with DPV analysis to determine the basal signal (Fig. S3). Then, our sensor responded to patient samples diluted as much as 2:1 × 10^5^ (610 copies/mL) with the overall regression equation of y = 11.321x + 29.512 (R^2^ = 0.973) (Fig. 5E,F). At end, sensor diluted as much as 1:4 × 10^5^ and sensed (457 copies/mL) when faced with specific antibody (Au@Anti-SARS-CoV-2 spike) in following the regression equation of y = 12.124x + 20.512 (R^2^ = 0.989) (Fig. 5G,H). Because of the various reagents and generates noise signals of VTM includes, we consider the LOD of the COVID-19 sensor to be low enough for practical use, for example ˂ 457. Our sensor can also screen and detect the SARS-CoV-2 virus from clinical samples without any preprocessing as using in point-of-care tests. Our results are summarized with other biosensors which they used for direct detection of surface antigen or whole viruses of SARS-CoV-2 in the Table 1.

**Table 1:** Comparison the biosensor methods to detection of surface antigen of SARS-COV2.

Sample volume^1^	Detection target	Detection method	Sensitivity (true positive rate^2^)	Specificity (true negative rate^3^)	Assay detection time	Real samples	References
	SARS-COV-2 spike S1	Gr-FET	0.2 pM	–	About 2 min	–	Tan et al.^28^
	SARS-COV-2 spike S1	Gr-FET	2.4 × 10^2^ copies/mL	–	> 1 min	Swab samples/cultured viruses	Mahari et al.^13^
	SARS-COV-2 spike S1	MV-gal1@Au/Anti-SARS-CoV-2	4.57 × 10^2^ copies/mL		> 5 min	Swab samples	This study

^1^The sensitivity of a clinical test refers to the ability to correctly identify those patient samples (also called the true positive rate) (Lalkhen and McCluskey^30^).

^2^The specificity of a clinical test refers to the ability to correctly identify those non-patient samples (also called true negative rate) (Lalkhen and McCluskey^30^).

### Reproducibility and stability of MV-gal1@Au/Anti-SARS-CoV-2 spike to sense the SARS-CoV-2 virus on the SCPE-GNP

The virus concertation of 1 × 10^5^ (virus particle/mL^−1^) relate to the MV-gal1 sensing and 1:4 × 10^5^ (virus particle/mL^−1^) relate to the Au/Anti-SARS-CoV-2 spike, was showed the standard deviation of 4.9% and 5.3% examined for five measurements, respectively with a showing good reproducibility (Fig. S4a). Our results also show the perfect response after 35 successively scanning, suggesting the acceptable durability of this method (Fig. S4b). Calibration results were reported in (Table S1) for MV-gal1@SARS-CoV-2 Antigen@Au/Anti-SARS-CoV-2 spike sensors.

### Clinical validation of MV-gal1@Au/Anti-SARS-CoV-2

To improve validation of clinical samples based on our sensor, percent positive agreement (PPA) and negative percent agreement (NPA) were calculated. Real time test included (E gene and N2 gene) was used by Emad laboratory. Our sensed samples by MV-gal1@Au/Anti-SARS-CoV-2 achieved a PPA of 96.38% (93% CI 93.64–95.96%) and NPA 98.46% (98% CI 93.84–99.00%) (Table S3). Two false negative sample by MV-gal1@Au/Anti-SARS-CoV-2 Were positive by Real time PCR. On the hand, one positive sample by MV-gal1@Au/Anti-SARS-CoV-2 was negative by Real time PCR.

### Statement on human guidelines

The clinical samples used in this were collected who suspicious patients referred to Emad laboratory were used. Ethical committed of Iran medical university confirmed that all experiments were performed in accordance with relevant guidelines as the declaration of Helsinki and regulations with registration code as: 99-1-6-8-17943. We also confirmed that experimental protocols were approved by Iran University of medical science and with financial committee by including a statement in the methods section to this effect, including any relevant details. We explained about human samples in section “Clinical sample preparation” and Table S2.

### Consent for publication

The graphic figure was prepared by Dr. Elham Ghazizadeh.

### Statement confirming

All our results have been achieved realistically and with great effort during the Corona pandemic in IRAN and our research were carried out in compliance with the ARRIVE guidelines.

## Conclusion

In the development of the COVID-19 pandemic, it is necessary to design the sensors to enhance screening and specific detection for COVID-19 in a short time^31,32^. For the first time, we designed a dual-sensor based on MV-gal1/Au@Anti-SARS-CoV-2 spike to detection SARS-CoV-2 Antigen. We used of the MV-gal1 to general screening of any spike virus. We introduce the natural bed-receptor (MV-gal1) with electrochemical reactions in solid sensor with can bind to glycan of any virus. At the end, using the Au@Antibody-SARS-CoV-2 spike cause to specific bind with the SARS-CoV-2 Antigen. So, our dual-platform has the ability of screening for any spike virus (at the first step) and specific detection of SARS-CoV-2 with high sensitivity (second step) in the solid biosensor. However, there are need more in vivo studies with more sample size to validate this sensor for another spike virus and SARS-CoV-2.

## Supplementary Information

Supplementary Information.
