# Supplementary material for: Dual electrochemical sensing of spiked virus and SARS-CoV-2 using natural bed-receptor (MV-gal1)
Source: Sci Rep. 2021 Nov 26;11:22969. doi: 10.1038/s41598-021-02029-0 (PMC8626484; doi:10.1038/s41598-021-02029-0)
Supplement: Supplementary file 1 — Supplementary Information. [file 41598_2021_2029_MOESM1_ESM.docx]

**Dual Electrochemical Sensing of Spiked virus and SARS-CoV-2 Using Natural Bed-receptor (MV-gal1)**

E. Ghazizadeh ^a,b^*, Ali Neshastehrizd,^b^ Ali Dehghani Firoozabadi^c^, E.Saievar-Iranizad^d^

aDepartment of Medical Biotechnology, School of Medicine, Mashhad University of Medical Sciences, Mashhad, Iran

bRadiation Biology Research Center, Iran University of Medical Sciences (IUMS), Tehran, Iran

c Yazd Cardiovascular Research Center, Shahid Sadoughi University of Medical Sciences,Yazd, Iran

d Department of Physics, Faculty of Science, University of Tarbiat Modarres, Tehran, Iran


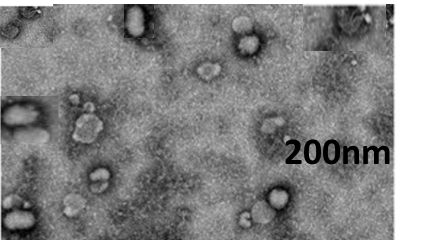


**Fig S1.** The images of TEM about characterization of MV-extracted from bone marrow-derived mesenchymal stem cells


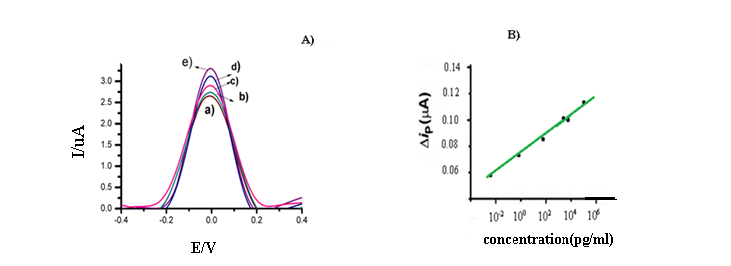

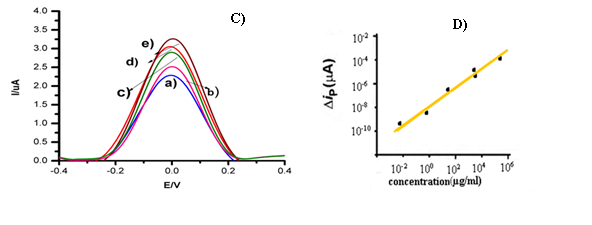


**Fig S2**. The performance of the sensor for screening and specific detection of SARS-CoV-2 in0.01× UTM(A) Screening of SARS-CoV-2 Antigen based on DPV images using the 500 ng/mL(e) to 1 µ/mL(a) on the MV-gal1/SCPE-GNP. (B) A calibration plot of the current density vs log concentration of SARS-CoV-2 Antigen. (C) Specific detection of SARS-CoV-2 Antigen based on DPV images using the 1 µg/mL (e) to 250 500fg/mL. (D) A calibration plot of the current density vs log concentration of SARS-CoV-2 Antigen.


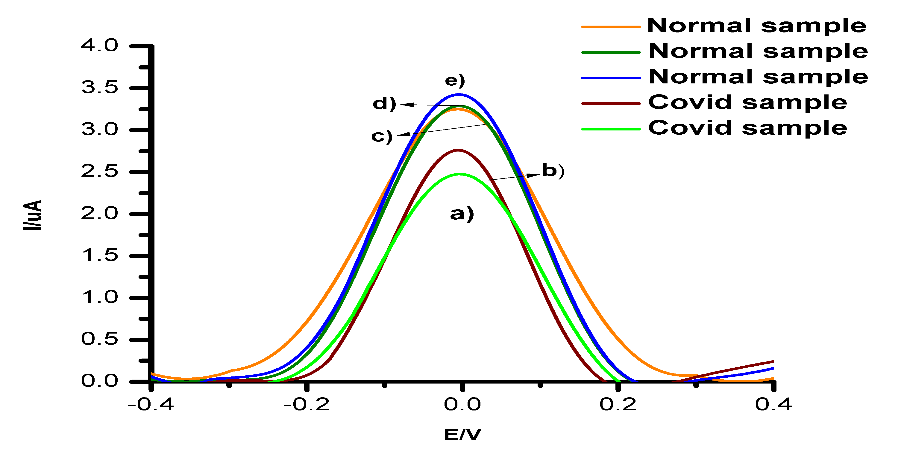


**Fig S3**. Normalize the sensor between the covid-19 samples and normal samples.


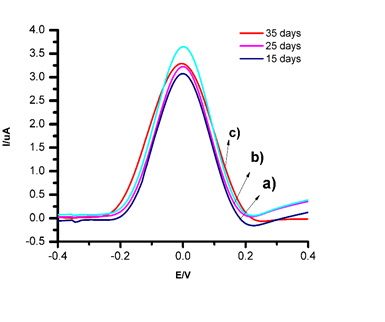


**A)**


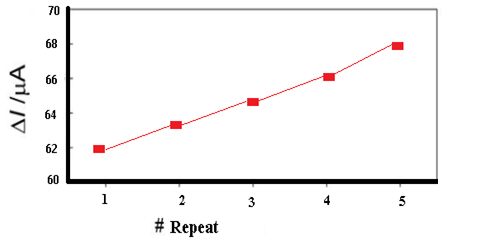


**B)**


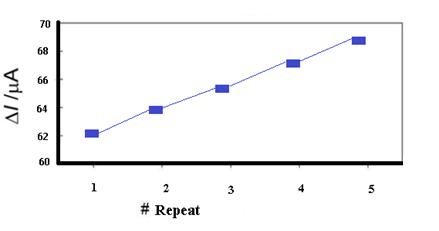


**C)**

**Fig S4.** (A) DPV behaviors of stability of MV-gal1@ SARS-CoV-2 Antigen@ Au/Anti-SARS-CoV-2 spike sensors in different days. (B, C) Reproducibility behaviors of MV-gal1@ SARS-CoV-2 Antigen@ Au/Anti-SARS-CoV-2 spike sensors while adding the SARS-CoV-2 Antigen and Au/Anti-SARS-CoV-2 spike.

**Table S1**. Clinical characteristics of laboratory confirmed patients with COVID-19

| patient#1 patient#2 patient#3 |  | patient#4 |
| --- | --- | --- |
| Age (years)/Sex 25/M 48/M 55/F  Fever + + +  Chills - - -  Cough + + +  Dyspnea - -  Diarrhea - - +  Viral load 3.12 × 10^6^ 2.23 × 10^6^ 3.14 × 10^5^ |  | 32/M  +  -  +  +  +  3.67× 10^4^ |

**Table S2**. Calibration results for MV-gal1@ SARS-CoV-2 Antigen@ Au/Anti-SARS-CoV-2 spike sensors

| Variable | Range tested | Selected Value |
| --- | --- | --- |
|  |  |  |
| Number of repetitions | 1-10 | 1-10 |
| Incubation time of SARS-CoV-2 Antigen and MV-gal1 (hour) | 18-24 | 24 |
| Incubation time of SARS-CoV-2 Antigen and MV-gal1 (temparture) | 25-37° | 26° |
| Anti-SARS-CoV-2 spike , uM | 25-37° | 34° |
| Dropping Anti-SARS-CoV-2 spike (temparture) | 4° | 4° |
| Dropping of SARS-CoV-2 Antigen (time) | 2-4 | 4 |

**Table S3.** Validation of MV-gal1@Au/Anti-SARS-CoV-2 Biosensor using clinical samples validated by Real-time PCR

| Real time PCR  MV-gal1@Au/Anti-SARS-CoV-2 Positive Negative Total  Biosensor |
| --- |
|  |
| Positive 4 1 5 |
| Negative 2 10 12 |
| Total 6 11 17  PPA 96.38 % (93% CI 93.64%–95.96%)  NPA 98.46 % (98 % CI 93.84%–99.00%) |
|  |

**
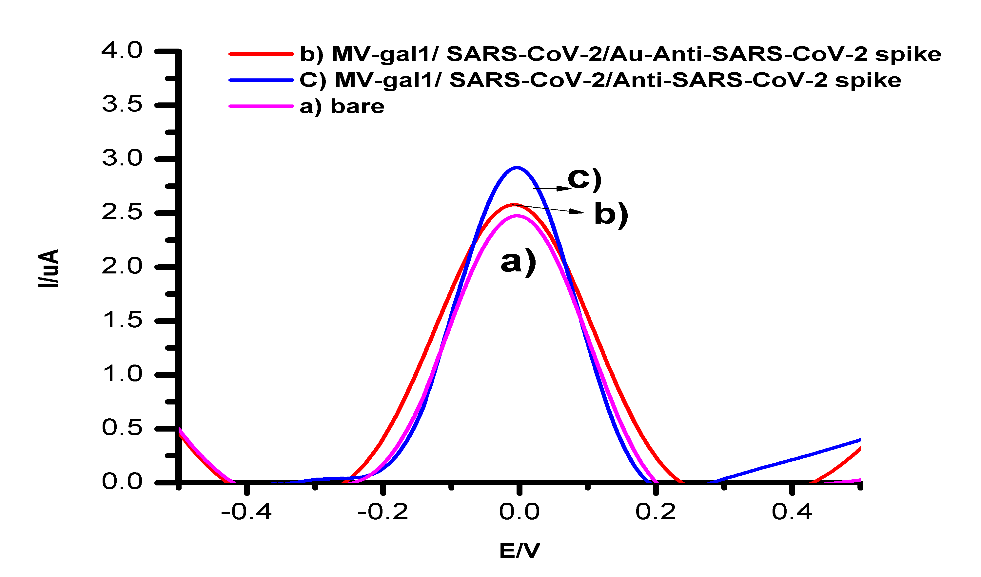
**

**Fig S5.** DPV behaviors for confirming the attachment o of MV-gal1@ SARS-CoV-2 Antigen@ Au/Anti-SARS-CoV-2 spike relate to MV-gal1@ SARS-CoV-2 Antigen@ Anti-SARS-CoV-2 (Without AuNP as control)
